# Determinants of vaccine hesitancy and effectiveness of vaccination counseling interventions among a sample of the general population in Palermo, Italy

**DOI:** 10.1080/21645515.2020.1728157

**Published:** 2020-03-18

**Authors:** Claudio Costantino, Francesca Caracci, Mariarosa Brandi, Stefania Enza Bono, Antonio Ferro, Claudia Emilia Sannasardo, Francesco Scarpitta, Andrea Siddu, Carlotta Vella, Gianmarco Ventura, Francesco Vitale, Alessandra Casuccio, Vincenzo Restivo

**Affiliations:** a Department of Health Promotion Sciences, Maternal and Infant Care, Internal Medicine and Medical Specialties (PROMISE), University of Palermo, Palermo, Italy; b Prevention Department, AULSS 6 Euganea, Padova, Italy; c Prevention Department, APSS Trento, Trento, Italy

**Keywords:** Vaccine hesitancy, vaccination counseling, health advocacy, general population, web search, internet traffic indicators, vaccination confidence

## Abstract

Counteract vaccine hesitancy is a public health priority. Main objectives of the cross-sectional study conducted were to evaluate knowledge, attitudes, and behaviors regarding vaccination issues, to estimate the prevalence of vaccine hesitancy and to estimate the effectiveness of vaccination counseling on community advocacy in a sample of general population. An anonymous validated questionnaire was administered in April 2017 at the main shopping center of Palermo and was followed by tailored vaccination counseling interventions. To estimate the effectiveness of the interventions four main connection parameters to the vaccinarsi.org website were evaluated, in the two months before and after the intervention and in the two months before the intervention compared with the same period of previous and following years. Among the 299 subject enrolled 12.7% were hesitant about vaccinations, and 4.7% declared being against vaccinations. General practitioners (GPs) and pediatricians were the most affordable source of information about vaccinations. A higher probability of vaccination hesitancy/refusal was reported among subjects who considered “alternative strategies” the best way for the prevention of infectious diseases (adj-OR = 7.01, IC95% 2.88–17.09, *p*-value < 0.001). A considerable increase of all the vaccinarsi.org website indicators analyzed was observed, from the area in which survey participants lived. Prevalence of vaccine hesitancy among population surveyed is consistent with the literature data. HCWs, such as GPs and pediatricians, should play a key role in modifying personal convictions and choices about vaccinations. A proper vaccination counseling could improve attitudes regarding vaccination issues, such as quality of web-based research.

## Introduction

Vaccination counseling is widely recognized by health authorities and the medical community as a major tool for counteracting vaccination hesitancy and for increasing health advocacy among the general population.^1^

Also a proper counseling programme is essential in changing people’s perceptions, knowledge and concerns about vaccination.^2^

Recently, the increasingly widespread use of the Internet among the general population to collect information regarding health and medical treatment has led to the circulation of fake news and many anecdotal documents on the effectiveness and safety of vaccines.^3^

As a consequence, the recent decline in vaccination coverage in Italy can be associated with the dissemination of incorrect information in the mass media and social media.^4,5^

Several studies showed that parents frequently acquire information on vaccination through the mass media and social media, focusing on potential collateral effects and adverse reactions.^6,7^

Around 15% of the general population of 13 Countries representative of all six World Health Organization (WHO) Regions was recently estimated as being hesitant about vaccinations, representing a priority target for tailored counseling intervention for the public health authorities.^8^ Additionally, healthcare workers (HCWs) play an important role in motivating and empowering patients and the general population about vaccination themes.^9^

Nevertheless, the HCWs themselves often exhibit a lack of confidence in vaccination strategies, such as influenza vaccination campaigns, and a lack of knowledge in providing proper counseling to the general population.^10-12^

The main objectives of the study were to evaluate knowledge, attitudes, and behaviors regarding vaccination issues and to estimate the prevalence of vaccine hesitancy in a sample of the general population in the Province of Palermo. Moreover, to estimate the effectiveness of the vaccination counseling intervention, the number of visits to vaccinarsi.org, their duration, and the number of new website visitors are evaluated in the two months before and after the interventions.

## Material and methods

A cross-sectional study was carried out in April 2017 at the Forum Palermo shopping center in Palermo by a team of nurses, public health medical residents, and public health doctoral students from the Hygiene and Preventive Medicine School of the University of Palermo, Italy.

All the team members were previously trained to limit the interviewer bias and the heterogeneity of data collection.

Palermo is the fifth most populous Italian province, with a population of 1,260,293 inhabitants.

### Questionnaire

An anonymous and previously validated questionnaire was administered to subjects who were randomly sampled among the people at the Forum Palermo shopping center on two different Saturdays (from 9:00 a.m. to 2:00 p.m.).^6^

Forum Palermo is the main shopping center in the city of Palermo and the most crowded, with approximately 20,000 daily visitors. The mall’s management estimates 6,000 people visit the shopping center on Saturday mornings.

To evaluate the representativeness of the sample, the basic numerousness of the sample to enlist (with a confidence interval of 95% and a desired precision of 5%) was previously calculated, corresponding to 196.

The participants were initially provided information that explained the goals of the study and the processing of personal data according to Italian privacy laws, as well as an informed consent form. If an individual agreed to participate and signed the informed consent form, the questionnaire was administered before the vaccination counseling intervention.

The questionnaire consists of 22 items, divided into four sections, as follows:^6^

- Demographic information and educational level, including gender, age, work activities, marital status, and parental status;
- Attitudes toward vaccinations included in the Sicilian immunization schedule both for infant and adult, where a positive attitude involved total acceptance of the vaccination offered, a hesitant attitude involved partial acceptance of the vaccination offered or a delay in its administration on the immunization schedule, and a negative attitude involved refusal of the vaccination included in the schedule;
- Knowledge and perceptions regarding vaccination and vaccine-preventable diseases;^6^
- Information sources on vaccination and the main vaccination-themed websites consulted and considered reliable.

Tailored counseling, in case of any doubt regarding the Sicilian vaccination schedule, vaccine-preventable diseases and related vaccines, and possible or suspected adverse reactions to vaccinations, was provided face to face by healthcare professionals and members of the study group. The mean duration spent for the individual counseling was around 15 minutes.

In addition, the participants all received informative flyers, shopper bags, balloons, and a copy of the Sicilian immunization schedule referring to VaccinarSì’s website, vaccinarsi.org.

The study was approved by the Palermo Ethical Committee 1 (session no. 12, December 2016).

### Analysis of vaccinarsi.org visits

To estimate the effectiveness of the intervention strategy, visits to vaccinarsi.org were matched with a georeferenced analysis by Google Analytics.

The website was created by members of the Italian Society of Hygiene, Preventive Medicine and Public Health to inform and educate the general population and the healthcare community about vaccinations and to oppose the anti–vaccination movement’s spread on the Internet.^13^

Social network accounts (e.g., on Facebook, Instagram, and Twitter), launched between 2013 and 2014, are also associated with VaccinarSì’s main web portal (vaccinarsi.org).

VaccinarSì’s website is organized into five main sections (vaccine-preventable diseases, registered vaccines, benefits and risks of vaccinations, the prevention of vaccine misinformation, travel immunization).

Its contents are validated by a scientific committee that includes more than 20 Italian and international experts on immunization from academia and Italian National Health Services.

Internet traffic from Palermo and Sicily (all the Sicilian cities in which survey participants lived were included in the analysis) to the VaccinarSì website was evaluated.

Four main indicators were taken into account:

- The number of unique visits to vaccinarsi.org,
- The number of new visitors,
- The number of pages viewed in each visit,
- The duration of each visit.

These parameters were all compared between the two months before the first counseling activity (from 8 February to the 7 April 2017) and the two months afterward (from 8 April to the 7 June 2017).

Moreover, average number of new unique visits to vaccinarsi.org in the two months after the interventions conducted (from 8th April 2017 to 7th June 2017) was compared with the same period of the two previous (2015, 2016) and following (2018, 2019) years.

### Statistical analysis

Absolute and relative frequencies were calculated for the categorical (qualitative) variables, and quantitative variables were summarized by their means (standard deviations). The differences in the categorical variables for hesitancy or refusal and between before and after the intervention were analyzed using chi-squared tests (Mantel–Haenszel) and the Student test for the means.

All the variables found to have a statistically significant association with vaccination hesitancy/refusal in the univariate analysis were included in a multivariate backward stepwise logistic regression model.

All variables with a *p*-value ≤ 0.20 were selected in the multivariate model, to guarantee a more conservative approach.

The crude odds ratio (crude OR) and the adjusted OR (adj-OR) with 95% confidence intervals (CIs) were also calculated in the logistic regression model. The level of significance chosen for the multivariate logistic regression analysis was 0.05 (two tailed).

We entered all the information into a database created with EpiInfo 3.5.4 (Centers for Disease Control and Prevention, Atlanta, USA). All the data were analyzed using the statistical software package Stata/MP 12.1 (StataCorp LP, College Station, TX, USA).

## Results

### Questionnaire analysis

Table 1 summarizes the sociodemographic and occupational characteristics of the surveyed population. A total of 299 adults (63.2% women) answered the questionnaire.

**Table 1.** Socio-demographic and occupational characteristics of the surveyed population (n = 299)

	n	%
**Gender**		
*- Male*	110	36.8
*- Female*	189	63.2
**Age, mean ± DS**	40 ± 14.2	
**Education**		
-*Secondary or lower*	67	22.4
-*High School degree*	232	77.6
**Marital status**		
*-Married/cohabitant*	232	77.5
*-Non married/single*	67	21.5
**Being parent**		
*-Yes*	235	78.6
*-No*	64	21.4
**Working in health sector**		
*-Yes*	43	14.4
*-No*	256	85.6
**Profession**		
*-Housewife*	77	25.8
*-Unemployed*	25	8.4
*-Student*	34	11.4
*-Elderly*	24	8
*-Job holder/freelancer*	139	46.4

The mean age was 40 years (standard deviation ±14.2 years). In 77.6% of cases, the education level was a high school degree, and only 14.4% of the surveyed sample worked in the health sector.

The majority of the sample participants (n = 235) were parents (78.6%).

Table 2 shows the attitudes of the surveyed population toward vaccinations. A total of 82.6% of participants in the sample (n = 247) had a positive attitude about vaccinations, denoting a willingness to receive vaccinations in the future, 12.7% (n = 38) were hesitant about vaccinations, and 4.7% (n = 14) declared being against vaccinations.

**Table 2.** Attitudes and sources of information about vaccination of the surveyed population and their confidence on the Public Health authorities recommendation (n = 299)

	n	%
**Best strategy for infectious disease prevention**		
*- vaccination*	269	90.0
*- other (hygiene, physical activity, homeopathy, etc.)*	30	10.0
**Attitude toward vaccinations**		
*- positive*	247	82.6
*- hesitant*	38	12.7
*- reluctant*	14	4.7
**Intention to get vaccinated in future**		
*-Yes*	247	82.6
*-No*	52	17.4
**Intention to vaccinate your own children in future (n = 222)**		
*-Yes, totally*	186	83.8
*-Yes, only with mandatory vaccinations*	22	9.9
*-No*	14	6.3
**Main information source on vaccinations (multiple answer)**		
*- General practitioners or Pediatricians*	243	81.3
*- Vaccination Center Health Professionals*	48	16.1
*- Relatives and Friends*	29	9.7
*-Mass media and internet*	72	24.0
*- Institutional websites*	45	15.1
**Websites previously consulted about vaccination issues (n = 244)**		
*- vaccinarsi.org*	45	18.5
*- focus.it*	67	27.4
*- Institutional websites (epicentro.iss.it e salute.gov)*	122	50.0
*- Anti–vaccination websites (mednat.org, comilva.org, disinformazione.it)*	10	4.1
**Reliable websites on vaccination issues (n = 224)**		
*- vaccinarsi.org*	49	21.9
*- focus.it*	44	19.6
*- Institutional websites (epicentro.iss.it e salute.gov)*	125	55.8
*- Anti–vaccination websites (mednat.org, comilva.org, disinformazione.it)*	6	2.7
**Greater confidence in HCWs than in mass-media/internet on vaccination topics (n = 297)**		
*- Yes*	259	87.2
*- No*	38	12.8
**HCWs failed to mention some vaccine-related adverse effects during counseling (n = 294)**		
*- Yes*	130	44.2
*- No*	164	55.8

At the same time, 83.8% of the subjects stated they would vaccinate their own children in the future according to the regional immunization schedule.

On the other hand, 9.9% of the parents interviewed said they would vaccinate their own children only with mandatory vaccinations, and 6.3% stated they would refuse future vaccinations for their children. Table 2 also presents the main sources of information on vaccinations and general perceptions about and trust in healthcare professionals.

General practitioners (GPs) and pediatricians were the main source of information in 81.3% of cases, followed by vaccination center workers (16.1%).

Official websites (15.1%), the mass media, and nongovernmental websites (24%) represented alternative sources of information about vaccinations. The questionnaire also asked which websites were consulted the most on vaccination issues and which were considered more reliable.

The most consulted websites on vaccination themes were institutional websites (50%), followed by focus.it (27.4%, a website with evidence based contents), and vaccinarsi.org (18.5%).

The most reliable websites were the institutional ones (55.8%), followed by vaccinarsi.org (21.9%) and focus.it (19.6%). Anti–vaccination websites seemed to have little visibility among the general population (4.1%) and were considered unreliable (2.7%).

Finally, 87.2% of the participants in the sample trusted the information provided by HCWs more than the information reported on the web (12.8%). However, 44.2% of the interviewees thought that the HCWs failed to mention some vaccine-related adverse effects during counseling.

Table 3 shows the results of the univariate/multivariate analysis. A higher probability of vaccination hesitancy/refusal was reported among subjects who considered the mass media, the Internet, and friends or relatives the most reliable source of information (crude OR = 2.09, IC95% 1.01–4.41, *p*-value = 0.05), among subjects who had no confidence in HCWs regarding vaccination topics (crude OR = 2.18, IC95% 1.02–4.75, *p*-value = 0.05), and, finally, among subjects who considered hygiene, physical activity, or homeopathy, instead of vaccinations, the best strategies for the prevention of infectious diseases (crude OR = 7.39, IC95% 3.32–16.44, *p*-value < 0.001), a number that was also significant in the multivariate analysis (adj-OR = 7.01, IC95% 2.88–17.09, *p*-value < 0.001).

**Table 3.** Univariate (crude OR) and Multivariate (adj-OR)* analysis between vaccination hesitancy or refusal (vs vaccination confidence) with different categorical variables considered in the study. (* *multivariate analysis was performed only for variables with p ≤ .0.20 at the univariate).*

	Vaccination hesitancy or refusal (vs confidence)			
	crude OR (95% CIs)	p-value	adj-OR (95% CIs)	p-value
**Gender**				
*- Female*	*reference*	0.48		
*- Male*	1.82 (0.81–2.36)			
**Age classes**				
- ≤ 40 years	*ref*	0.15	*ref*	0.57
*- ≥ 40 years*	1.56 (0.85–2.56)		1.21 (0.61–2.37)	
**Education**				
*- high school/university degree*	*ref*	0.54		
*- primary/secondary school degree*	0.79 (0.37–1.68)			
**Marital Status**				
*- married/partner*	*ref*	0.67		
*- single/widower/divorced*	1.16 (0.58–2.33)			
**Being parent**				
*- yes*	*ref*	0.49		
*- no*	1.28 (0.64–2.58)			
**Health-care workers**				
*- yes*	*ref*	0.14	*ref*	0.19
*- no*	2.25 (0.76–6.59)		2.14 (0.69–6.67)	
**Best strategy for infectious disease prevention**				
*- vaccination*	*ref*	**<0.001**	*ref*	**<0.001**
*- other (hygiene, physical activity, homeopathy, etc.)*	**7.39 (3.32–16.44)**		**7.01 (2.88–17.09)**	
**Main informative source on vaccination topics**				
*- GPs, pediatricians, public health-care workers*	***ref***	**0.05**	*ref*	0.19
*- Mass media, social networks, friends/relatives*	**2.09 (1.01–4.41)**		1.73 (0.77–3.89)	
**Confidence in HCWs regarding vaccination topics**				
*- yes*	*ref*	**0.05**	*ref*	0.98
*- no*	**2.18 (1.02–4.75)**		0.99 (0.38–2.54)	
**Healthcare Professionals failed to mention some vaccine-related events**				
*- yes*	*ref*	0.21		
*- no*	1.47 (0.80–2.68)			

### Analysis of connection data to vaccinarsi.org website

**Figure 1.**
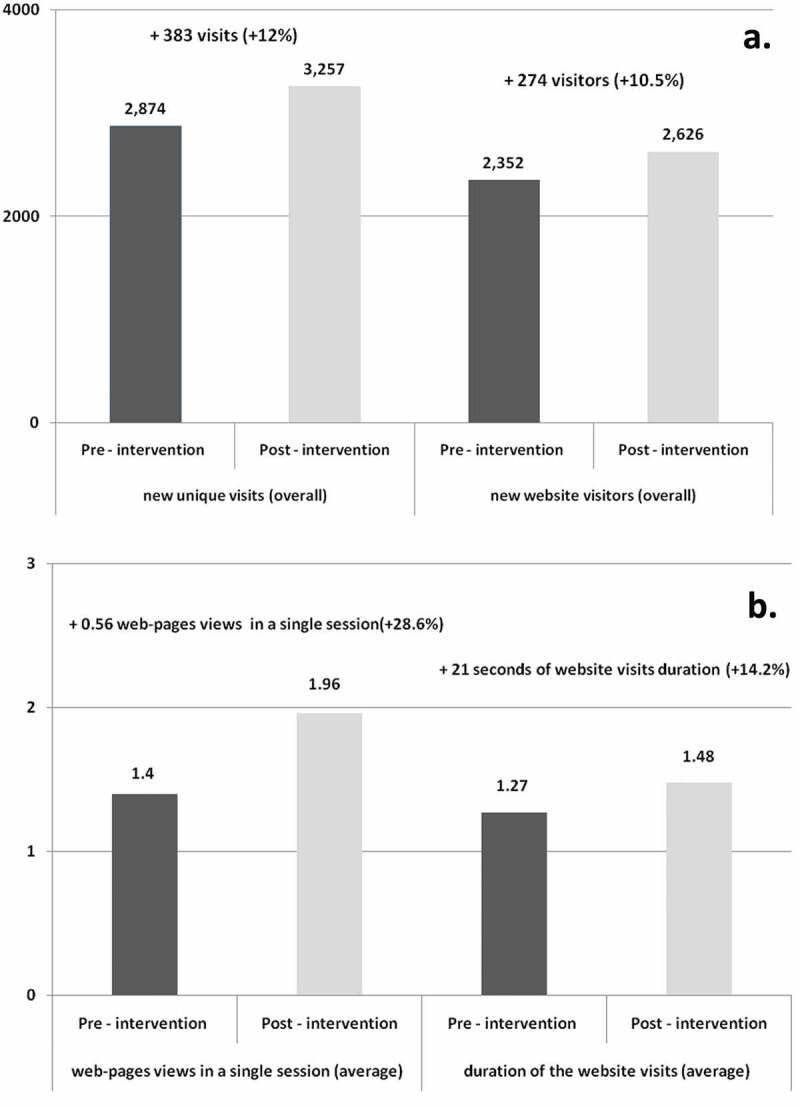
Comparison of new unique visits, new website visitors (a), average webpages views in a single session and average duration of the website vaccinarsi.org visits in minutes (b), in the two months before (from 8th February 2017 to 7th April 2017) and after (from 8th April 2017 to 7th June 2017) vaccination counseling conducted at the principal shopping center of Palermo. (data limited to all the Sicilian cities in which survey participants lived)

Figure 1(a,b) compares the number of visits to vaccinarsi.org between the two months before and the two months after the intervention. A 12% increase was noted in the number of new unique visits to vaccinarsi.org (+383, *p*-value < 0.01). A 10.5% increase (+274, *p*-value < 0.01) in the number of new website visitors was reported (Figure 1(a)).

Moreover, a significant increase in the average number of page views at vaccinarsi.org in a single session was reported (+0.56 pages per session, +28.6%, *p*-value < 0.05), and a concomitant increase (+21 seconds, +14.2%, *p*-value < 0.05) in the average duration of the website visits was observed (Figure 1(b)).

**Figure 2.**
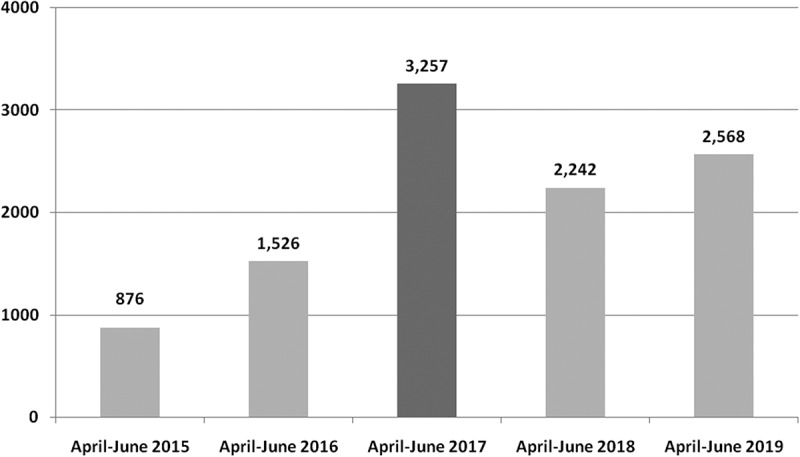
Average new unique visits to vaccinarsi.org in the two months after the interventions conducted (from 8th April 2017 to 7th June 2017), compared with the same period of the two previous (2015, 2016) and following (2018, 2019) years (data limited to all the Sicilian cities in which survey participants lived)

A comparison of the number of unique visits to vaccinarsi.org between the two months after the intervention and the same period of the two previous (2015, 2016) and following (2018, 2019) years was carried out. A constant increase of visits to vaccinarsi.org from April-June 2015 (n = 876) to the same period of 2019 (n = 2,568) is displayed in Figure 2.

In particular, in the period April-June 2017 (after intervention counseling) a consistent and significant increase of the number of unique visits to vaccinarsi.org (n = 3,257) in comparison with two years before and after is reported, from all the Sicilian cities in which survey participants lived.

## Discussion

Vaccine hesitancy refers to a delay in the acceptance of vaccines or their outright refusal, despite the availability of vaccination services. This phenomenon has recently become so vast and troubling that, in 2013, the World Health Organization (WHO) Strategic Advisory Group of Experts recommended an interview study with immunization managers in 13 countries to understand the main determinants of vaccine hesitancy worldwide.^8^

Because of the dynamic and changing nature of vaccine hesitancy, the factors influencing the phenomenon, such as sociodemographic features, perceptions and personal experiences, attitudes, and knowledge, must be continuously monitored.^14-16^

In our study, GPs and pediatricians were the most reliable and affordable sources of information about vaccination issues. Consequently, HCWs should improve knowledge about vaccine-preventable diseases and about vaccination counseling techniques with the general population and parents.^17,18^

Several studies conducted in Europe confirmed the key role of HCWs in modifying knowledge, personal convictions, and choices about vaccinations among the general population.^19-21^

In the univariate analysis, a significant association with a positive attitude regarding vaccination was observed among subjects who trusted the vaccine information provided by the HCWs.

The general population’s perceptions about the risk of vaccinating themselves or their children are often conditioned more strongly by emotive, cognitive, and social factors than by scientific data, and personal experience usually plays a role in parents’ acceptance or refusal of vaccinations.^22^ Moreover, widespread access to nongovernmental and unscientific websites and the mass media have created distrust among the population seeking information from alternative sources.

In the sample analyzed, 24% preferred consulting the mass media and the web about vaccination topics, as opposed to 15.1% of subjects who sought information from institutional websites. Generally, according to those surveyed, institutional websites were the most frequently consulted (50%) and were perceived as being the most reliable websites (55.8%).

Only 4.1% of the subjects consulted websites with a strong anti-scientific orientation (e.g. mednat.it or disinformazione.it), generating further disinformation and spreading false information such as the relation between measles, mumps and rubella (MMR) vaccines and autism or how alternative measures can be more effective than vaccinations in preventing infectious diseases.^23^

Relatives and friends can also be a source of information about vaccinations that is often incorrect, leading to possible negative effects on vaccination knowledge and on the personal decision to properly vaccinate.^24^ However, the survey results showed relatives and friends to be only a limited source of information (9.7%).

Subjects with a hesitant or reluctant attitude toward vaccination were those who considered the mass media, the Internet, and friends and relatives as their main sources of information on vaccination topics, as opposed to HCWs, or who considered hygiene, physical activity, or homeopathy, rather than vaccinations, the best strategies for infectious disease prevention.

Several studies previously showed that parents who are hesitant about vaccinations can differ in several sociodemographic characteristics related to education level and household income.

Parents who refuse vaccinations for their children frequently have low levels of trust in the government and healthcare professionals, and follow the recommendations of complementary or alternative medicine professionals, whom they consider to be reliable sources of vaccine information, similar to the observations in our population.^25,26^

The findings in this study can be useful in constructing a tailored strategy to manage vaccine-hesitant parents by establishing a positive dialogue, providing education targeting their concerns, maintaining a relationship with these families, and making every effort to follow the recommended immunization schedule.

According to WHO, a multipronged approach and dialogue-based intervention appear to be one of the most effective strategies to address vaccine hesitancy.^27^

Major limitations, however, affect the present study. First, even though the sample reflects the same patterns of vaccination hesitancy/refusal as among the general population, a selection bias due to a lack a participation of those with negative attitudes toward vaccines should be taken into account.

Moreover, the shopping center, similarly to other Italian and European Cities, is far from the city center and near suburban neighborhoods of the town.

However, the possibility to reach the mall directly from city center with the tramway could limit the selection bias. Furthermore, the prevalence of vaccine hesitancy observed among our population is consistent with literature data, suggesting a good representativeness of the sample enrolled.^8^

In addition, the time elapsed between the study and the results presentation could modify the estimation of the vaccination attitudes or acceptance (that was a self-reported item to ensure anonymity of participants), but this bias especially regards short-term preventive attitudes and acute diseases with rapid changes. Vaccine hesitancy is a relatively stable phenomenon, because it involves a long-lasting preventive concern that rarely changes over time and refers to vaccine-preventable diseases with both short and long clinical evolutions.^8^

Finally, many other factors could contribute to the increase of the VaccinarSì’s website parameters analyzed in the study before and after the interventions conducted, such as the mass media/political level debate about the implementation of the Law 119/2017 that introduced ten mandatory vaccination for attending kindergarten.^28^

However, the georeferenced increase in both unique visits and new visitors to vaccinarsi.org during the two months after the interventions, could equally suggest a positive impact of the vaccination counseling provided after the administration of the questionnaire.

Other experiences showed that it could be possible to change some convictions and attitudes toward vaccinations, properly interfacing with the general population through correct communication and web-based strategies.^6^

A good starting point could be the proper channeling of requests for information about vaccinations on the Internet.^27,29^

The aim of counseling is not to suggest, persuade, or even direct but, overall, to encourage a better and more conscious vaccine awareness and greater acceptance. The necessity of accessing vaccine-hesitant subjects through a planned communicative strategy to eliminate their reluctance has already been underlined.^30^

To promote a vaccination culture among the general population, also vaccination-related knowledge ant attitudes of HCWs must be improved.^31^

As previously reported, HCWs usually play an important role in vaccination counseling, both for subjects with a positive attitude and for subjects with a hesitant/reluctant attitude toward vaccinations.^27^

Only with tailored training in communicative vaccination strategies can the personal attitudes and counseling strategies of HCWs on vaccination topics be improved, making them public health opinion leaders who will provide the general population accurate information and lead to a community health advocacy about vaccination issues.
